# Improved Confidence Intervals of a Small Probability from Pooled Testing with Misclassification

**DOI:** 10.3389/fpubh.2013.00039

**Published:** 2013-10-07

**Authors:** Chunling Liu, Aiyi Liu, Bo Zhang, Zhiwei Zhang

**Affiliations:** ^1^Department of Applied Mathematics, Hong Kong Polytechnic University, Hong Kong, PR China; ^2^Biostatistics and Bioinformatics Branch, Eunice Kennedy Shriver National Institute of Child Health and Human Development, Rockville, MD, USA; ^3^Biostatistics Core, School of Biological and Population Health Sciences, Oregon State University, Corvallis, OR, USA; ^4^Division of Biostatistics, Center for Devices and Radiological Health, Food and Drug Administration, Silver Spring, MD, USA

**Keywords:** confidence intervals, coverage probability, exact inference, pooling, prevalence, rare event, sensitivity, specificity

## Abstract

This article concerns construction of confidence intervals for the prevalence of a rare disease using Dorfman’s pooled testing procedure when the disease status is classified with an imperfect biomarker. Such an interval can be derived by converting a confidence interval for the probability that a group is tested positive. Wald confidence intervals based on a normal approximation are shown to be inefficient in terms of coverage probability, even for relatively large number of pools. A few alternatives are proposed and their performance is investigated in terms of coverage probability and length of intervals.

## 1 Introduction

Screening for subjects infected with a disease can be costly and time-consuming, especially when the disease prevalence is low. In an effort to overcome these barriers, Dorfman (1) proposed the pooling of blood samples to test for syphilis antigen. According to his procedure, blood samples from subjects under screening are pooled prior to testing. If a pool of blood samples is tested negative, then all subjects in the pool are declared free of infection. Otherwise, a positive test result on a pool indicates that at least one subject is infected and retesting of all individuals in that pool is then conducted to find the infected subjects.

Since its appearance, Dorfman’s (1) pooled testing (also known as group testing) approach has drawn considerable attention. The approach has been applied to other areas of screening (than syphilis), such as human immunodeficiency virus (HIV) testing [e.g., Westreich et al. (2)]. A number of variations have been developed, and the scope has been expanded to include estimation of the prevalence of a disease (without necessarily identifying the diseased individuals) (3–24). However, there is relatively little discussion on the possibility of misclassification (i.e., that the disease status of an individual or a pool of individuals can be assessed incorrectly because the biomarker may be imperfect).

The existing literature on estimating the prevalence of a disease using the pooled testing approach is focused on point estimation (3, 5–7, 13–15, 17, 20, 22, 23). Construction of confidence intervals for the prevalence of a disease has been discussed by Hepworth (10–12) and Tebbs and Bilder (21). These authors assumed that the disease status of a subject can be accurately determined, which may be unrealistic in practice. For example, Weiss et al. (25) reported 97.7% sensitivity and 92.6% specificity for detecting HIV infection with an enzyme-linked immunosorbent assay, and Deitz et al. (26) reported 94% sensitivity for determining the status of *N*-acetyltransferase 2 with a commonly used 3-single nucleotide polymorphism genotyping assay.

This article focuses on construction of efficient confidence intervals for the prevalence of a rare disease using Dorfman’s pooled testing procedure when the disease status is determined by an imperfect biomarker subject to misclassification. We investigate the unified approach of Tu et al. (27), which produces a confidence interval for the disease prevalence by converting a confidence interval for the probability of a pool being tested positive. We then demonstrate that Wald confidence intervals based on a normal approximation are inefficient in that they have a repetitive up-and-down behavior in the coverage probability, similar to that of the classical normal approximation binomial confidence interval discovered by Brown et al. (28). In the present context, this up-and-down behavior persists even when the number of pools is relatively large. We derive alternative confidence intervals by extending the methods of Wilson (29), Clopper and Pearson (30), Agresti and Coull (31), and Blaker (32). Simulation studies are conducted to compare the performance of the proposed methods in terms of coverage probability and mean length. The methods are applied to a real example concerning the seroprevalence of HIV among newborns.

## 2 Interval Estimation Under Pooled Testing

Suppose one wants to estimate the prevalence of a disease in a population, *p* = *P*(*D* = 1), where *D* denotes the disease status of a subject in the population, with *D* = 1 if the subject is infected with the disease. We assume that the disease status is determined using an imperfect biomarker *M*, taking values 0 and 1, and a subject is classified as infected if *M* = 1. The accuracy of the biomarker is measured by its specificity π_0_ = *P*(*M* = 0|*D* = 0) and sensitivity π_1_ = *P*(*M* = 1|*D* = 1). For the biomarker to be of practical use we assume that 1/2 < π_0_, π_1_ ≤ 1; otherwise a random assignment of the disease status would perform better than the biomarker.

Supposed a random sample of size *nk*, where *n* and *k* are positive integers, is available from the target population. The conventional approach to estimating *p* is based on individually observed values of *M*, say *M*_1_, …, *M_nk_*. Dorfman’s procedure for estimating *p* is carried out by randomly assigning the *nk* individuals into *n* pools with *k* individuals in each pool and testing for positivity of the biomarker for each pool. Inference on *p* is then based on the number of pools that are tested positive. For this purpose, further testing for biomarker positivity for each individual in the pool is not necessary. Thus, instead of observing *M*_1_, …, *M_nk_*, the biomarker values of the *k* individuals in a pool, we observe $\tilde{M}=\max\text{\{}M_{1},\;\cdots \;,M_{k}\text{\}}$. If $\tilde{M}=0$ , then *M_i_* = 0 for each *i* = 1, …, *k*. If $\tilde{M}=1$ , then *M_i_* = 1 for at least one *i* in the pool. Throughout we assume that pooling will not affect the misclassification of the disease status by the biomarker. Let $\delta =P\left(\tilde{M}=1\right)$ be the probability that a pool is tested positive. Then it follows from Tu et al. (23) that

(1) $$\delta =\pi_{1}-r{\left(1-p\right)}^{k},\;\;\left(r=\pi_{0}+\pi_{1}-1\right).$$

Consequently,

$$p=1-{\left(\frac{\pi_{1}-\delta }{r}\right)}^{1∕k}.$$

For fixed *k*, π_0_, and π_1_, the value of δ as a function of *p* increases as *p* increases. Because 0 ≤ *p* ≤ 1, we have

(2) $$1-\pi_{0}\leq \delta \leq \pi_{1}.$$

Using the relationship given by equation (1) along with the constraint equation (2), a unified (and straightforward) approach (27) to constructing a confidence interval for *p* is as follows. Suppose [δ_*L*_, δ_*U*_] is a confidence interval for δ with level 1 − α. Define

(3) $$p_{L}=1-{\left(\frac{\pi_{1}-\delta_{L}}{r}\right)}^{1∕k},\;\;p_{U}=1-{\left(\frac{\pi_{1}-\delta_{U}}{r}\right)}^{1∕k}.$$

Then [*p*_*L*_, *p*_*U*_] is a confidence interval for *p* with level 1 − α.

## 3 Constructing Confidence Intervals for δ

In this section we propose a few methods to construct a confidence interval for δ. Once derived, the interval can then be converted into a confidence interval for *p*, as indicated in the previous section. Let ${\tilde{M}}_{i}$, *i* = 1, …, *n*, be the test result for the *i*th pool. The ${\tilde{M}}_{i}$ are independent and identically distributed Bernoulli variables with $P\left({\tilde{M}}_{i}=1\right)=\delta \in \left[1-\pi_{0},\pi_{1}\right]$. Thus a confidence interval for δ can be constructed using methods developed for a binomial probability. However the constraint equation (2) must be taken into account. In what follows we extend several popular methods for binomial confidence intervals to construct confidence intervals for δ, taking the constraint equation (2) into consideration.

### 3.1 The Wald confidence interval

The Wald confidence interval is based on the fact that the estimator of δ, $\hat{\delta }=S∕n$, is asymptotically normally distributed with mean δ and variance δ(1 − δ)/*n*, where $S=\sum_{i=1}^{n}\;{\tilde{M}}_{i}$ is the number of pools that are tested positive. Without the constraint equation (2), the Wald confidence interval is given by

$$\tilde{I}=\hat{\delta }\pm Z_{1-\alpha ∕2}{\left\{\hat{\delta }\left(1-\hat{\delta }\right)\right\}}^{1∕2}∕n^{1∕2},$$

where *Z*_1 − α/2_ is the 100(1 − α/2)th percentile of the standard normal distribution. With the constraint, we define the Wald confidence interval for δ as

(4) $$I=\tilde{I}⋂\left[1-\pi_{0},\pi_{1}\right],$$

where

$$\left[a_{1},b_{1}\right]⋂\left[a_{2},b_{2}\right]\equiv \left[\max\left(a_{1},a_{2}\right),\min\left(b_{1},b_{2}\right)\right].$$

### 3.2 The Wilson confidence interval

Without any constraints on the binomial probability, the Wilson confidence interval (29) is

$${\tilde{I}}_{W}=\frac{S+Z_{1-\alpha ∕2}^{2}∕2}{n+Z_{1-\alpha ∕2}^{2}}\pm \frac{Z_{1-\alpha ∕2}n^{1∕2}}{n+Z_{1-\alpha ∕2}^{2}}{\left\{\hat{\delta }\left(1-\hat{\delta }\right)+Z_{1-\alpha ∕2}^{2}∕\left(4n\right)\right\}}^{1∕2}\;.$$

Accounting for the constraint equation (2), the modified Wilson confidence interval for δ is given by

(5) $$I_{W}={\tilde{I}}_{W}⋂\left[1-\pi_{0},\pi_{1}\right].$$

### 3.3 The Clopper–Pearson confidence interval

The Clopper–Pearson confidence interval is often referred to as the exact confidence interval due to its derivation based on the binomial distribution rather than the normal approximation. Note that *S* follows a binomial distribution with size *n* and probability δ. Let *s* be the observed value of *S*. If there are no constraints on δ, then the lower bound δ_*L*_ and the upper bound δ_*U*_ of the Clopper–Pearson interval can be derived by solving the equations:

$$\begin{array}{llll} P_{\delta_{L}}\left(S\geq s\right) & =1-B\left(s-1;n,\delta_{L}\right)=\alpha ∕2,\;\;\text{and}\; & & \;\\ P_{\delta_{U}}\left(S\leq s\right) & =B\left(s;n,\delta_{U}\right)=\alpha ∕2,\; & & \; \end{array}$$

respectively, where *b*(*s*; *n*, δ) = *P*_δ_(*S* = s) is the binomial density function with size *n* and probability δ, and $B\left(s;n,\delta \right)=\sum_{i=1}^{s}\;b\left(s;n,\delta \right)=P_{\delta }\left(S\leq s\right)$ is the corresponding binomial distribution function. Tu et al. (27) suggested using this interval without any modification for δ. The modified Clopper–Pearson confidence interval that accounts for the constraint equation (2) is given by

(6) $$I_{\mathrm{CP}}=\left[\delta_{L},\delta_{U}\right]⋂\left[1-\pi_{0},\pi_{1}\right].$$

### 3.4 The Agresti–Coull confidence interval

The Agresti–Coull confidence interval is a modification of the Wald confidence interval with $\hat{\delta }$ replaced by

$$\tilde{\delta }=\frac{S+Z_{1-\alpha ∕2}^{2}∕2}{n+Z_{1-\alpha ∕2}^{2}}.$$

Thus, when δ is not constrained, the Agresti–Coull confidence interval is given by

$${\tilde{I}}_{AC}=\tilde{\delta }\pm Z_{1-\alpha ∕2}{\left\{\tilde{\delta }\left(1-\tilde{\delta }\right)\right\}}^{1∕2}∕n^{1∕2}.$$

With the constraint equation (2), the Agresti–Coull confidence interval becomes

(7) $$I_{AC}={\tilde{I}}_{\mathrm{AC}}⋂\left[1-\pi_{0},\pi_{1}\right].$$

### 3.5 The Blaker confidence interval

The confidence intervals of Wilson, Clopper–Pearson, and Agresti–Coull are highly recommended by Brown et al. (28). Blaker (32) proposed a method to improve the standard “exact” confidence intervals for discrete distributions, and called the resulting confidence intervals *acceptability intervals*. For the binomial case, the author showed that the acceptability interval is shorter than the Wald, Wilson, and Agresti–Coull intervals. Define

$$\gamma \left(\delta ,s\right)=\min\left\{1-B\left(s-1;n,\delta \right),B\left(s;n,\delta \right)\right\}$$

and

$$\alpha (\delta ;\;s)=\sum_{\left\{i:\;\gamma (\delta ,i)\leq \gamma (\delta ,s)\right\}}b(s;n,\delta ).$$

Then for the binomial probability δ with no constraints, by reformulating the notation of Blaker (32), the Blaker interval is given by ${\tilde{I}}_{B}=\left\{\delta :\;\alpha \left(\delta ;s\right)>\alpha \right\}$. Blaker (32) showed that ${\tilde{I}}_{B}$ is indeed an interval and has coverage probability 1 − α. When δ is constrained by equation (2), the Blaker confidence interval can be defined as

(8) $$I_{B}={\tilde{I}}_{B}⋂\left[1-\pi_{0},\pi_{1}\right].$$

## 4 Simulations

There has been a large amount of research on the performance of various binomial confidence intervals for the disease prevalence *p* under the usual setting where independent and identically distributed Bernoulli observations of the disease status are available. However, not much research has been conducted under Dorfman’s pooled testing setting, especially in the presence of misclassification. In this section we conduct simulations to compare the coverage probability and mean length of the confidence intervals for *p*, and to investigate the effect of the pool size *k* and the misclassification rates (i.e., 1 − π_0_ and 1 − π_1_) on the precision (coverage and length) of the intervals. It is worth noting that when a confidence interval [δ_*L*_, δ_*U*_] for δ is converted into a confidence interval [*p*_*L*_, *p*_*U*_,] for *p* via equation (3), the coverage probability remains unchanged because of the monotonicity of *p* as a function of δ.

### 4.1 The oscillation behavior of Wald confidence intervals

Brown et al. (28) investigated the performance of a number of confidence intervals for a binomial probability in the usual setting, where the individual disease status is observed without error, corresponding to *k* = 1 and π_0_ = π_1_ = 1 in our setting. The authors showed a remarkable oscillation up-and-down behavior of the widely used Wald confidence intervals based on a normal approximation; the coverage probability of the interval increases from far below the nominal level of 1 − α to the nominal level and then decreases, and the pattern repeats until the sample size becomes rather large. We demonstrate here that Wald confidence intervals have the same oscillation up-and-down phenomenon under pooled testing with misclassification.

Fixing specificity π_0_ = 0.85 and sensitivity π_1_ = 0.90, we computed via simulation the coverage probability of the Wald confidence interval for *p*, in a variety of scenarios with *k* = 2, 5, 10 ≤ *n* ≤ 150, and *p* = 0.01, 0.10. For each configuration of (*k*, *n*, *p*), 10,000 simulations were conducted. For each simulation, we generated a random observation from the binomial distribution with probability δ = π_1_ − *r*(1 − *p*)*^k^* and size *n*, and constructed the 95% Wald confidence interval for δ according to equation (4). This confidence interval for δ was then converted into a confidence interval for *p* using equation (3). The average coverage probability of the confidence interval is the proportion of the 10,000 intervals that contain the true value of *p*.

**Figure 1:**
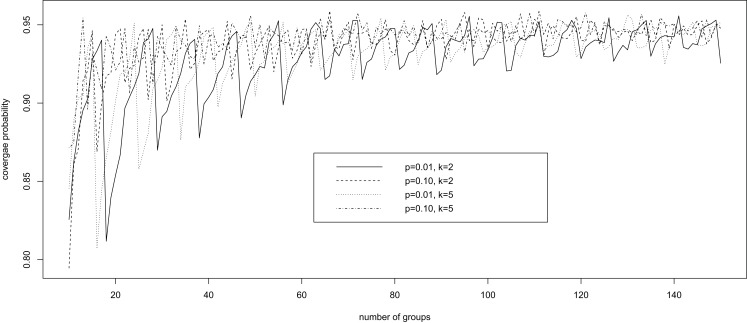
**The oscillation up-and-down behavior of the Wald confidence interval under pooled testing with misclassification**.

Figure 1 presents the simulation results. It is clear that, for each configuration, the coverage probability as a function of *n* starts with very low coverage, usually below 85%, and then gradually increases as *n* gets larger to a value near the nominal level of 95%. Then it quickly decreases to a low coverage probability. The trend then repeats until *n* is large enough to stabilize the coverage probability. Therefore, unless *n* is sufficiently large, the Wald confidence interval does not provide the desired coverage and should not be recommended. This unfortunate observation is consistent with that of Brown et al. (28) for the classical binomial confidence intervals.

### 4.2 Comparison of confidence intervals

Using simulations again we compared the precision of the four alternative confidence intervals, the Wilson, Clopper–Pearson, Agresti–Coull, and Blaker intervals, along with the Wald interval, in terms of mean length and coverage probability. To set up the simulation we considered various representative configurations of (*p*, *n*, *k*, π_0_, π_1_) with *p* = 0.001, 0.1, 0.3, *n* = 10, 20, 50, *k* = 2, 5, 10, and (π_0_, π_1_) = (0.85, 0.95). A total of 10,000 simulations were conducted, and the coverage probability of each interval was estimated the same way as for the Wald interval. The mean length of each interval was estimated by averaging over the 10,000 simulated intervals.

Table 1 shows the estimated overage probability and average length of each confidence interval in various scenarios with *p* = 0.001 (the results for *p* = 0.1 and *p* = 0.3 are similar and therefore not shown). In almost all cases considered, the four alternative confidence intervals (i.e., the Wilson, Clopper–Pearson, Agresti–Coull, and Blaker intervals) provide satisfactory coverage probability around the 95% nominal level of confidence. The Wald intervals are quite unstable, with poor precision when *n* is small. The Clopper–Pearson and Agresti–Coull intervals tend to be more conservative, producing higher coverage probability, followed by the Blaker interval and then the Wilson interval. However, conservatism usually comes with the price of longer intervals, as shown in Table 1.

**Table 1:** **Empirical comparison of confidence intervals in terms of coverage probability and average length (see Section 4.2 for details)**.

*k*	π_0_	π_1_	Empirical coverage					Average length				
			Wald	Wilson	C-P	A-C	Blaker	Wald	Wilson	C-P	A-C	Blaker
***n* = 10**												
2	0.85	0.85	0.796	0.951	0.989	0.989	0.951	0.162	0.252	0.297	0.285	0.270
2	0.85	0.95	0.791	0.951	0.989	0.989	0.951	0.136	0.214	0.250	0.240	0.229
2	0.95	0.85	0.407	0.911	0.987	0.987	0.987	0.061	0.204	0.233	0.221	0.230
2	0.95	0.95	0.412	0.908	0.987	0.987	0.987	0.054	0.179	0.204	0.194	0.201
5	0.85	0.85	0.804	0.946	0.990	0.990	0.946	0.077	0.115	0.139	0.132	0.123
5	0.85	0.95	0.802	0.948	0.990	0.990	0.948	0.063	0.095	0.113	0.108	0.102
5	0.95	0.85	0.424	0.901	0.986	0.986	0.986	0.029	0.089	0.104	0.098	0.101
5	0.95	0.95	0.425	0.903	0.986	0.986	0.986	0.025	0.077	0.089	0.084	0.087
10	0.85	0.85	0.808	0.939	0.989	0.989	0.939	0.044	0.061	0.076	0.072	0.065
10	0.85	0.95	0.809	0.940	0.989	0.989	0.940	0.034	0.050	0.060	0.057	0.053
10	0.95	0.85	0.449	0.980	0.980	0.980	0.980	0.017	0.047	0.055	0.052	0.053
10	0.95	0.95	0.452	0.982	0.982	0.982	0.982	0.014	0.040	0.047	0.045	0.046
***n* = 20**												
2	0.85	0.85	0.825	0.978	0.978	0.978	0.978	0.118	0.164	0.181	0.172	0.172
2	0.85	0.95	0.820	0.978	0.978	0.978	0.978	0.101	0.141	0.154	0.147	0.148
2	0.95	0.85	0.651	0.918	0.980	0.980	0.980	0.051	0.123	0.132	0.127	0.137
2	0.95	0.95	0.652	0.922	0.983	0.983	0.983	0.046	0.109	0.117	0.112	0.121
5	0.85	0.85	0.826	0.974	0.974	0.974	0.974	0.052	0.071	0.079	0.075	0.075
5	0.85	0.95	0.836	0.974	0.993	0.974	0.974	0.045	0.061	0.067	0.064	0.064
5	0.95	0.85	0.655	0.981	0.981	0.981	0.981	0.022	0.052	0.056	0.054	0.058
5	0.95	0.95	0.671	0.979	0.979	0.979	0.979	0.021	0.047	0.050	0.048	0.052
10	0.85	0.85	0.835	0.974	0.993	0.974	0.974	0.027	0.037	0.041	0.039	0.039
10	0.85	0.95	0.846	0.972	0.993	0.972	0.972	0.024	0.032	0.035	0.033	0.033
10	0.95	0.85	0.698	0.972	0.994	0.972	0.972	0.013	0.028	0.030	0.029	0.031
10	0.95	0.95	0.700	0.973	0.995	0.973	0.973	0.011	0.024	0.026	0.025	0.027
***n* = 50**												
2	0.85	0.85	0.943	0.957	0.974	0.957	0.957	0.074	0.093	0.098	0.095	0.096
2	0.85	0.95	0.942	0.953	0.972	0.953	0.953	0.065	0.081	0.085	0.082	0.083
2	0.95	0.85	0.925	0.955	0.986	0.955	0.955	0.038	0.064	0.066	0.063	0.069
2	0.95	0.95	0.927	0.956	0.986	0.956	0.956	0.034	0.057	0.059	0.056	0.061
5	0.85	0.85	0.937	0.945	0.968	0.945	0.945	0.032	0.040	0.042	0.040	0.041
5	0.85	0.95	0.946	0.949	0.971	0.949	0.949	0.028	0.035	0.036	0.035	0.035
5	0.95	0.85	0.933	0.949	0.983	0.983	0.983	0.017	0.027	0.028	0.027	0.029
5	0.95	0.95	0.933	0.948	0.983	0.983	0.983	0.015	0.024	0.025	0.024	0.026
10	0.85	0.85	0.901	0.947	0.967	0.967	0.947	0.017	0.021	0.022	0.021	0.021
10	0.85	0.95	0.901	0.947	0.971	0.971	0.971	0.015	0.018	0.019	0.018	0.018
10	0.95	0.85	0.941	0.974	0.974	0.974	0.974	0.009	0.014	0.015	0.014	0.015
10	0.95	0.95	0.803	0.974	0.993	0.974	0.974	0.008	0.013	0.013	0.013	0.014

The effect of misclassification on the Wald interval does not seem to be clear due to its oscillation up-and-down behavior. For the other intervals, it appears that the coverage probability increases as the sensitivity π_1_ increases or as the specificity decreases. For fixed misclassification rates (π_0_, π_1_), including more samples in a pool seems to improve the coverage probability, up to certain pool size. This latter observation seems to agree with that of Tu et al. (23) and Liu et al. (33), who found that in presence of misclassification the efficiency of estimation increases with the pool size up to a certain point.

## 5 Example

We now illustrate the methods by applying them to a real example concerning the seroprevalence of HIV among newborns in the State of New York (34). The data were obtained by testing blood specimens from all infants born in this state during a 28-month period (from November 30, 1987 through March 31, 1990). The test was targeted at serum antibodies produced by the immune system in response to HIV infection. A positive test result indicates HIV infection in the mother but not necessarily in the child. To illustrate the methods, we focus on the Manhattan area, where 50,364 newborns were tested with 799 positive results.

Because the study did not involve pooled testing, we create pools in a *post hoc* manner by grouping subjects randomly into pools of a given size (*k* = 5 or 10). With *k* = 10, for instance, we obtain 5,036 pools of size 10, ignoring the four additional subjects. The test result for each pool is taken to be the maximum of all individual test results in the pool; that is, a pool is declared positive if and only if it contains one or more infants with positive test results. To account for possible misclassification, estimation of HIV seroprevalence requires knowledge of the sensitivity and specificity of the test. Because the true values of these performance measures are not known precisely, we perform a sensitivity analysis that covers a range of plausible values for the sensitivity and the specificity of the test. The reasoning of Tu et al. (27) and the numerical results in their Table 1 suggest that the specificity of the HIV test in this study is at least 99%. Accordingly, our sensitivity analysis includes the values 99, 99.5, and 99.9% for the specificity. The appears to be less information about the sensitivity of the HIV test in this study, and we therefore consider a wider range (95, 97.5, 99, and 99.9%) for the sensitivity. For each pair of sensitivity and specificity values and each value of *k*, we apply the five methods described earlier to the pooled dataset to obtain five 95% confidence intervals for the individual-level HIV seroprevalence rate, in addition to a point estimate (common to all five methods).

Table 2 presents the results of our sensitivity analysis (with different combinations of sensitivity and specificity values) for each value of *k* (5 or 10). It appears that the results are more sensitive to the specificity of the HIV test than to the sensitivity of the test. The point estimate and the confidence limits (for all five methods) tend to decrease with the sensitivity of the test and increase with the specificity of the test, as predicted by theory. Intuitively, increased sensitivity means fewer false negatives, and increased specificity means fewer false positives, and these are reflected in the estimates in Table 2. Between the different pool sizes (5 and 10), which lead to different datasets, there are some numerical differences, especially at lower values of the sensitivity and the specificity. However, when the sensitivity and the specificity are both high (say, 99.9%), there is remarkable agreement between the estimates based on *k* = 5 and those based on *k* = 10. In any case, the five confidence intervals are generally similar to each other, perhaps as a result of the large sample size.

**Table 2:** **Analysis of HIV seroprevalence data (see Section 5 for details)**.

Specificity	Sensitivity	Pt. est.	95% Confidence interval for *p* (%)									
π_0_ (%)	π_1_ (%)	$\hat{p}$ (%)	Wald		Wilson		Clopper–Pearson		Agresti–Coull		Blaker	
***k* = 5**												
99	95	1.48	1.36	1.60	1.36	1.60	1.36	1.60	1.36	1.60	1.36	1.60
99	97.5	1.44	1.32	1.55	1.33	1.56	1.33	1.56	1.32	1.55	1.33	1.56
99	99	1.42	1.30	1.53	1.31	1.53	1.30	1.53	1.30	1.53	1.31	1.53
99	99.9	1.40	1.29	1.51	1.29	1.52	1.29	1.52	1.29	1.51	1.29	1.52
99.5	95	1.58	1.46	1.70	1.47	1.70	1.47	1.70	1.46	1.70	1.47	1.70
99.5	97.5	1.54	1.43	1.66	1.43	1.66	1.43	1.66	1.43	1.66	1.43	1.66
99.5	99	1.52	1.40	1.63	1.41	1.63	1.41	1.63	1.40	1.63	1.41	1.63
99.5	99.9	1.50	1.39	1.61	1.39	1.62	1.39	1.62	1.39	1.61	1.39	1.62
99.9	95	1.67	1.55	1.78	1.55	1.79	1.55	1.79	1.55	1.78	1.55	1.79
99.9	97.5	1.62	1.51	1.74	1.51	1.74	1.51	1.74	1.51	1.74	1.51	1.74
99.9	99	1.60	1.48	1.71	1.49	1.71	1.49	1.71	1.48	1.71	1.49	1.71
99.9	99.9	1.58	1.47	1.69	1.47	1.70	1.47	1.70	1.47	1.69	1.47	1.70
***k* = 10**												
99	95	1.59	1.47	1.71	1.47	1.72	1.47	1.72	1.47	1.71	1.47	1.72
99	97.5	1.55	1.43	1.67	1.43	1.67	1.43	1.67	1.43	1.67	1.43	1.67
99	99	1.52	1.41	1.64	1.41	1.64	1.41	1.64	1.41	1.64	1.41	1.64
99	99.9	1.51	1.39	1.62	1.40	1.62	1.39	1.63	1.39	1.62	1.40	1.62
99.5	95	1.64	1.52	1.77	1.53	1.77	1.52	1.77	1.52	1.77	1.53	1.77
99.5	97.5	1.60	1.48	1.72	1.48	1.72	1.48	1.72	1.48	1.72	1.48	1.72
99.5	99	1.57	1.46	1.69	1.46	1.69	1.46	1.69	1.46	1.69	1.46	1.69
99.5	99.9	1.56	1.44	1.67	1.45	1.67	1.44	1.67	1.44	1.67	1.44	1.67
99.9	95	1.69	1.56	1.81	1.57	1.81	1.57	1.81	1.56	1.81	1.57	1.81
99.9	97.5	1.64	1.52	1.76	1.53	1.76	1.52	1.76	1.52	1.76	1.52	1.76
99.9	99	1.61	1.50	1.73	1.50	1.73	1.50	1.73	1.50	1.73	1.50	1.73
99.9	99.9	1.60	1.48	1.71	1.49	1.71	1.48	1.71	1.48	1.71	1.48	1.71

## 6 Discussion

In this article we proposed a few approaches to constructing a confidence interval for the disease prevalence under pooled testing with misclassification. These approaches share a common feature in that they are all obtained by converting a valid confidence interval for the probability of a pool being tested positive. Our investigation of the coverage probability and mean length of the confidence intervals indicates that caution needs to be taken in using the Wald interval when the sample size is not large enough. From our overall evaluation it appears that the Clopper–Pearson and Agresti–Coull intervals, though somewhat conservative, tend to be more valid than the Wilson and Blaker intervals, especially when the disease probability and the sample size are relatively small.

Misclassification of the disease status clearly impacts the precision of the confidence intervals, as demonstrated by the simulation results in Figure 1 and Table 1. In this article, the misclassification is assumed to be independent of the pool size, which seems to be a reasonable assumption in some situations. However, as noted by Cahoon-Young (35), this assumption may be violated when the pool size gets larger. It remains to be seen how the performance of a confidence interval might be affected by pool-size-dependent misclassification.

## Conflict of Interest Statement

The authors declare that the research was conducted in the absence of any commercial or financial relationships that could be construed as a potential conflict of interest.
